# Immune-epigenetic crosstalk in haematological malignancies

**DOI:** 10.3389/fcell.2023.1233383

**Published:** 2023-09-21

**Authors:** Hera Wong, Ryohichi Sugimura

**Affiliations:** School of Biomedical Sciences, Lee Ka Shing Faculty of Medicine, The University of Hong Kong, Pokfulam, Hong Kong SAR, China

**Keywords:** leukemia, cancer, stem cell, niche, epigenetics

## Abstract

Haematological malignancies comprise a diverse set of lymphoid and myeloid neoplasms which can arise during any stage of haematopoiesis in the bone marrow. Accumulating evidence suggests that chronic inflammation generated by inflammatory cytokines secreted by tumour and the tumour-associated cells within the bone marrow microenvironment initiates signalling pathways in malignant cells, resulting in activation of master transcription factors including Smads, STAT3, and NF-κB which confer cancer stem cell phenotypes and drive disease progression. Deciphering the molecular mechanisms for how immune cells interact with malignant cells to induce such epigenetic modifications, specifically DNA methylation, histone modification, expression of miRNAs and lnRNAs to perturbate haematopoiesis could provide new avenues for developing novel targeted therapies for haematological malignancies. Here, the complex positive and negative feedback loops involved in inflammatory cytokine-induced cancer stem cell generation and drug resistance are reviewed to highlight the clinical importance of immune-epigenetic crosstalk in haematological malignancies.

## 1 Introduction

Haematopoietic stem cells (HSCs) are multipotent tissue stem cells which reside in the bone marrow (BM) and maintain the life-long generation of mammalian blood cells. This process, namely haematopoiesis, is tightly regulated by intrinsic features of HSCs, as well as extrinsic factors from BM microenvironment (Orkin and Zon, 2008). The BM microenvironment is a complex amalgamation of various cell types, both haematopoietic and non-haematopoietic, the extracellular matrix (ECM), chemokines, cytokines and physical factors. These cells and factors work in tandem to achieve an equilibrium between HSC quiescence and activation, as well as determining cell fate of proliferation, self-renewal, or differentiation. Dysregulation of BM architecture, for instance, perturbation of HSC differentiation, occurs mainly due to acute and chronic infections or ageing, and can result in immature progenitor cells of either the myeloid or lymphoid lineages. Subsequently, this leads to neoplastic transformation into one of the three large groups of haematological malignancies: leukaemia, lymphoma, and myeloma (Mendelson and Frenette, 2014; SanMiguel et al., 2020).

The classical model of haematological malignancies suggests that somatic mutations in genes encoding for signalling proteins or cell cycle regulatory proteins are required for neoplastic transformations. For instance, the nuclear factor-κB (NF-κB), Janus kinase (JAK)-signal transducer and activator of transcription (STAT), and extracellular signal-regulated kinase (ERK) pathways (Vainchenker and Constantinescu, 2012). A recent *de novo* characterisation study of acute myeloid leukaemia (AML) suggests that more than 40% of patients do not bear mutations in signalling genes, with only 22% bearing transcription factor mutations, and less than 50% displaying gene fusion and translocation events (Duployez et al., 2016). Likewise, somatic mutations in the RAS pathway and haematopoiesis regulatory genes are only present in 67% and 58% of paediatric T cell acute lymphoblastic leukaemia (T-ALL) cases (Zhang et al., 2012), while less than 50% of multiple myeloma (MM) patients harbour mutations in the MAPK/ERK pathway and DNA repair pathways (Heider et al., 2021). These findings suggest that epigenetics also plays a key role in regulating haematopoiesis and leukaemogenesis.

Somatic mutations are not the only molecular mechanism which initiates tumorigenesis in haematological malignancies. Rather, both molecular mechanisms work in tandem to contribute towards tumour progression, originally shown as DNA hypomethylation by Feinberg and Vogelstein (Feinberg and Vogelstein, 1983). For instance, JAK2 directly regulates histone modification and oncogenesis. JAK2 is present in the nuclei of HSCs and phosphorylates histone H3, and its upregulation leads to the expression of Lmo2, a known oncogene in leukaemia (Dawson et al., 2009). Epigenetic mechanisms which regulate haematopoiesis and leukaemogenesis in HSCs will be discussed further below.

Not only do genetic and epigenetic mechanisms intertwine during neoplastic transformation, the BM microenvironment also plays a crucial role in the acquisition of malignant phenotype of HSCs and therapy resistance. Emerging studies have shown that cells within the BM niche can interact with tumour cells through various cell-cell mechanism, chemokines and cytokines and induce epigenetic changes in regulatory pathways in a crosstalk manner, favouring processes such as migration, metastasis, metabolic reprogramming and hijacking of stem cell abilities by cancer stem cells (CSCs) (Wainwright and Scaffidi, 2017). Addressing these aspects can contribute to a better understanding of the BM, more importantly, mechanisms underlying chemotherapy and immunotherapy resistance of haematological malignancies, which can allow more robust screening for new therapeutic targets.

This review aims to summarise the regulatory factors involved in haematopoiesis within the BM and then address the two fundamental hallmarks of cancer: tumour microenvironment (TME) and epigenetic alterations in haematological malignancies to explore how they relate and favour, together, leukaemogenesis, progression, and resistance in haematological malignancies. In the final section, the clinical implications based on this immune-epigenetic crosstalk are also discussed.

### 1.1 Bone marrow microenvironment in haematopoiesis and its malignancy

**FIGURE 1:**
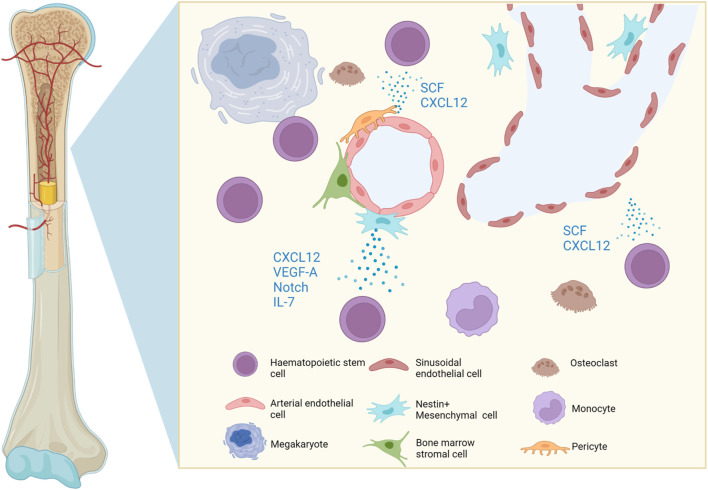
Graphic representation of the cells and soluble molecules present in the bone marrow microenvironment. The perivascular niche favours the uptake of oxygen and nutrients, whilst cells and soluble factors regulate HSC homing, mobilisation, quiescence, self-renewal and lineage commitment. Created with BioRender.com.

The BM is a multifunctional tissue which contains stem, progenitor and mature cells of multiple lineages. HSCs are multipotent tissue stem cells which reside in the bone marrow (BM) and maintain the life-long generation of mammalian blood cells through differentiation into either myeloid, lymphoid or erythroid lineages (Orkin and Zon, 2008). In contrast, BM cells are of mesenchymal origin, namely mesenchymal stromal cells. The self-renewal of HSCs and differentiation into blood-cell lineages are strictly regulated by multiple extrinsic and intrinsic factors within the BM microenvironment, for instance, niche-associated cytokines, chemokines, growth factors, transcription factors, and chromatin modifiers (Figure 1).

The BM microenvironment harbours HSCs and regulates their homing, mobilisation, quiescence, self-renewal or lineage commitment. HSCs reside in the perivascular niche, a functionally structured zone composed of endothelium, pericytes, mesenchymal stromal cells and sympathetic nerves (Morrison and Scadden, 2014). The endothelial cells are closely associated with perivascular mesenchymal stromal cells and both play a key role in maintaining and regulating HSCs. For instance, endothelial cells produce SCF and CXCL12 to maintain HSCs (Ding et al., 2012; Ding and Morrison, 2013). In addition, mesenchymal stromal cells also produce CXCL12, vascular endothelial growth factor A (VEGF-A) (Kobayashi et al., 2010), Notch ligands and growth factors to maintain the stem-cell pool and regulate self-renewal of HSCs, whereas secretion of cytokine IL-7 promotes B lymphopoiesis (Butler et al., 2010). The endothelium is a heterogeneous niche, where both sinusoidal and arterial endothelial cells work in tandem to maintain HSCs (Acar et al., 2015; Itkin et al., 2016).

The perivascular niche maintains haematopoiesis and promotes the expansion of HSCs in response to BM damage. The essence of normal HSC niche is to tightly regulate their resident number by cytokines and chemokines. However, leukaemic cells expand without limit while they co-occupy the HSC niche. This implies either leukaemic cells are ignorant of limiting factors from the niche, or they actively remodel the niche. Studies have shown that leukaemic blasts can remodel the BM niches and transform them into a malignant leukaemic microenvironment (Schepers et al., 2015). Monocytes are the predominant immune cell type in the tumour, not a mere filler (Cassetta and Pollard, 2018). Indeed, they actively support the survival and growth of leukaemic cells. Vasculature also plays a critical role here, where leukaemic cells interact and provoke inflammatory programs in endothelial cells. Subsequently, inflammation signals from endothelial cells differentiate classical monocytes to a non-classical phenotype, the latter of which support leukaemic cells (Witkowski et al., 2020), allowing leukaemic cells to remodel the niche and support themselves as well in the dynamic BM microenvironment. These examples illustrate that whilst factors in the BM microenvironment work together to tightly regulate hematopoiesis, their dysregulation through either somatic or epigenetic alterations can change the BM landscape and initiate tumorigenesis.

### 1.2 Epigenetics in haematopoiesis and its malignancy

Epigenetics are indispensable in haematopoiesis and leukaemogenesis. Epigenetics refers to changes to the chromatin structure and the accessibility of transcriptional machinery to DNA, hence modulating gene expression without altering the DNA sequence. Multiple pathways are involved in chromatin remodelling and epigenetic regulation, such as DNA methylation, histones modifications, and non-coding RNAs (ncRNAs) (Allis and Jenuwein, 2016). Whilst epigenetic mechanisms are highly flexible and play a crucial role in cell development, stem cell renewal, genome integrity and proliferation, their dysregulation is central to tumorigenesis and confer resistant phenotypes in cancer cells.

For instance, DNMT3a establishes DNA methylation during development, hence called *de novo* DNA methyltransferase. Somatic mutations in DNMT3a locus are recurrent in leukaemia, suggesting its role in haematopoiesis. DNMT3a plays an essential role in HSC differentiation, where its deletion was shown to block HSC differentiation, and the resultant HSCs expanded and accumulated in the body (Challen et al., 2011). Further study revealed DNMT3A-deficient HSCs can self-renew and expand over 12 generations of serial murine transplantation (Jeong et al., 2018). These findings indicate that DNMT3A-mediated DNA methylation curbs HSC expansion and limits leukaemogenesis.

MYC also plays a critical function in regulating haematopoiesis and leukaemogenesis. MYC has conserved long enhancers around the gene. Genomic analysis of T cell acute lymphoblastic leukaemia (T-ALL) cohorts identified the amplification of the MYC enhancer, which is functionally required for the development of T cells in the thymus. Mechanistically, NOTCH protein binds with the enhancer and induces T cell proliferation. This study sheds light on the fact that NOTCH, a well-known T cell regulator epigenetically regulates both haematopoiesis and leukemogenesis (Herranz et al., 2014). In addition, another MYC enhancer is essential for HSC function and leukaemogenesis via the fusion gene MLL-AF9 (Bahr et al., 2018), also indicating the importance of epigenetic regulation of MYC to haematopoiesis and leukemogenesis.

Post-transcriptional modification of RNAs also influences stem cell fate and oncogenesis, which is well documented in miRNA genesis (Ha and Kim, 2014). Dicer1 is a master regulator of miRNA genesis and its mutation is implicated in multiple cancers. Deletion of Dicer1 in mesenchymal progenitor cells in BM induced myeloproliferative disease and leukaemia pre-disposition, resembling the Shwachman-Bodian-Diamond syndrome (Raaijmakers et al., 2010). Moreover, Adenosine-to-inosine (A-to-I) editing of RNA is also essential for embryonic development. Deletion of its regulator ADAR1 was shown to result in foetal lethality. Mechanistically, RNA editing distinguishes them from pathogenic RNA. Thus, loss of ADAR1 renders its own RNA as “non-self” and provokes a lethal interferon reaction in the body (Liddicoat et al., 2015). In HSCs, ADAR1 responds to cytokine-driven STAT signalling and edits miRNA let-7. A-to-I editing impairs let-7 biogenesis, inducing unopposed upregulation of oncofetal protein LIN28B that results in leukaemic stem cell expansion (Zipeto et al., 2016). Taken together, these studies show that DNA methyltransferase, enhancers of oncogenes as well as post-transcriptional RNA modification play a critical role in leukaemogenesis and haematopoiesis, and that their dysregulation contributes towards haematological malignancies.

### 1.3 Microenvironment in haematopoiesis and diseases

In addition to intrinsic epigenetic alterations in HSCs, the BM microenvironment also releases cytokines and determines cellular phenotype through epigenetic changes (Figure 1). Emerging studies have shown that cytokines can activate the transcription of epigenetic modifiers and confer a CSC phenotype in cancer cells, hence promoting disease progression and treatment resistance in haematological malignancies in a crosstalk manner. For instance, immunocompromised mice transplanted with human CD34^+^ cells which were transduced with the MLL-AF9 fusion protein, a primary translocation event t(9;11) (p22; q23) commonly observed in leukaemias, were shown to develop into different types of leukaemia: acute myeloid leukaemia (AML), acute lymphocytic leukaemia (ALL), or biphenotypic leukaemia depending on the cytokines in the culture medium. Though the epigenetic or secondary genetic alterations were not elucidated in this study, it suggests that the BM microenvironment is required for the progression and fate determination of haematological malignancies, either via epigenetic alterations or secondary passenger mutations (Raaijmakers et al., 2010).

Moreover, haematological malignancies harbour particularly heterogeneous cell populations compared to solid tumours when exposed to chemotherapy and radiotherapy treatments. Other than resistance-conferring somatic mutations within certain cell subsets, non-mutational mechanisms of drug resistance through epigenetic modifiers are also commonly observed in a small population of “cancer stem cells” (CSCs) (Lapidot et al., 1994; Bonnet and Dick, 1997). CSCs are defined by their capacity to initiate cancer in immune deficient hosts in transplant, and are more refractory to the toxicity of chemotherapy and radiotherapy due to their intrinsic self-renewal abilities (Boutzen et al., 2022). Signalling derived from the pro-inflammatory microenvironment contributes greatly to their phenotype. An inflammatory state in haematological malignancies is often favoured by the release of pro-inflammatory cytokines such as interleukin-1 (IL-1), interleukin-6 (IL-6), tumour necrosis factor α (TNF-α) by immune cells, which activate epigenetic modifiers to promote the cell cycle and avoidance of apoptosis and cancer cells (Caiado et al., 2021; Table 1).

**TABLE 1:** Overview of epigenetic alterations induced by cancer–immune cell communication.

Cancer	Immune regulator	Source(s) of immune regulator	Epigenetic mechanism	Effect on cancer cell	References
MM	IL-6	Myeloma cells (autocrine), bone marrow stromal cells	JAK/STAT3 pathway activating methylation of p53	Bypassing cell cycle progression checkpoints	Hodge et al. (2005)
MM	IL-6		IL-6/STAT3 triggering piRNA 823 expression and promoting global DNA methylation	Upregulation of self-renewal and CSC phenotype	Ai et al. (2019)
AML, CML	IL-6	myeloid cells and HSPCs (autocrine)	IL-6/STAT3 hyperactivation induces expression of lncRNA Morrbid which represses Bcl2l11	Increased survival and proliferation	Cai et al. (2018)
T-ALL	TGFβ	Nonmyelinating Schwann cells, megakaryocytes	Loss of TGFβ resulting in downregulation of SMAD pathway and RUNX1	Decreased myeloid cell survival and differentiation in HSC, shift to lymphoid lineage	Wolfraim et al. (2004), Himes et al. (2005), Quéré et al. (2014)
T-ALL	IL-17	Activated T helper 17 lymphocytes	NF-κB pathway and upregulation of MMP9	Increased migratory capacity and liver metastasis	Tu et al. (2019)
AML	TNFα	Activated macrophages, T lymphocytes and natural killer cells	NF-κB pathway/p65, upregulation of Birc3 and downregulation of CASP-8	Increased self-renewal and stemness	Zitvogel et al. (2015), Yamashita and Passegué (2019)

Abbreviations: AML, acute myeloid leukaemia; IL-6, interleukin-6; IL-17, interleukin-17; MM, multiple myeloma; T-ALL, T cell acute lymphoblastic leukaemia; TGFβ, Transforming growth factor β; TNFα, Tumour necrosis factor α.

#### 1.3.1 IL-6

An example of how cytokines can induce epigenetic changes and favour the transformation of one phenotype to another is interleukin-6 (IL-6) in multiple myeloma (MM), a neoplasm characterised by abnormal plasma cell development (Rajkumar and Kyle, 2005). Elevated levels of IL-6 in the serum of MM patients directly correlate with tumour burden and severity of disease (Stephens et al., 2012).

**FIGURE 2:**
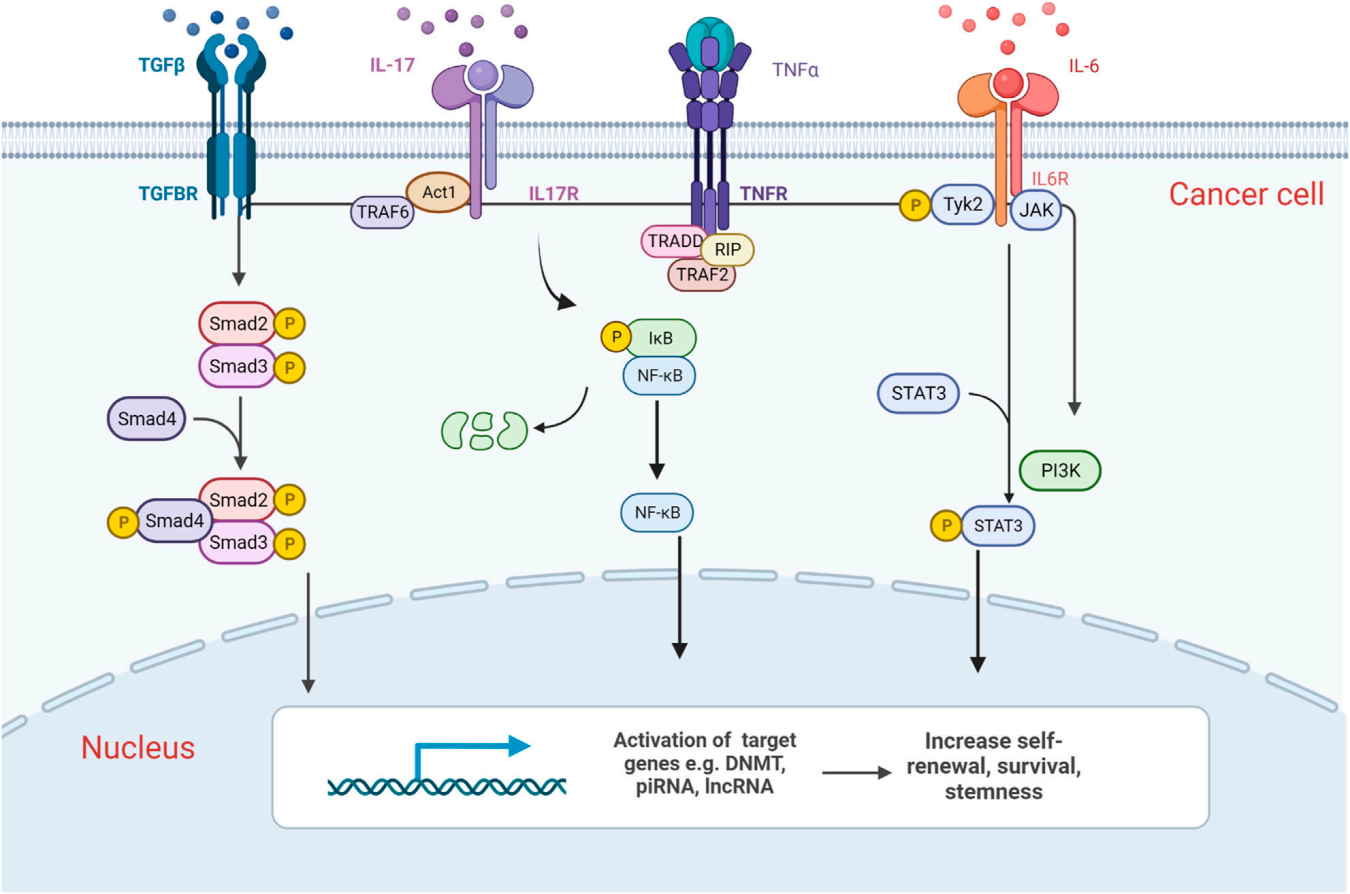
Graphic representation of epigenetic alterations induced by cancer–immune cell communication in haematological malignancies. Components of the BM microenvironment release cytokines which activate downstream signalling pathways such as NF-κB, SMAD and JAK/STAT, which in turn activates the transcription of chromatin modifiers. They induce epigenetic changes in cancer cells and transform their phenotype, increasing their self-renewal, survival and thereby promoting disease progression. Created with BioRender.com.

IL-6 is an inflammatory cytokine that activates signalling via three different modes. IL-6 binds to either the trans-membrane receptor IL-6Rα or to its soluble isoform sIL-6Rα, or mbIL-6Rα to form a complex and recruit the transmembrane-associated signal transducer gp130 (glycoprotein 130) to form a signal transduction complex. IL-6IL-6Rα/gp130 signalling then activates the intracellular signalling pathways JAK-STAT3, JAK-MAPK, and JAK-PI3K. JAK-mediated phosphorylation of STAT3, which leads to the formation of STAT3 homodimer complexes that translocate into the nucleus and to activate transcription of IL-6 target genes (Heinrich et al., 1998; Figure 2).

Myeloma progenitors in the BM microenvironment take advantage of both myeloma-cell-derived (autocrine) and tumour microenvironment-derived (paracrine) IL-6 released by non-malignant bystander cells such as bone marrow stromal cells (BMSCs). IL-6 signals via STAT3 and enhances the activity of the epigenetic modifier DNA methyltransferase 1, which drives CpG island methylation in promoter regions of p53 tumour suppressor gene, hence its deactivation. These epigenetic changes facilitate malignant MM cells to bypass cell cycle progression checkpoints and evade apoptotic signals resulting from DNA damage (Hodge et al., 2005), consequently promoting drug resistance and disease development in patients (Tu et al., 1998).

In addition, IL-6 also induces epigenetic changes to contribute to the stemness of cancer cells. For instance, a study showed that co-culture of human MM cells with IL-6-releasing polymorphonuclear myeloid-derived suppressor cells (PMN-MDSCs) resulted in increased relative expression of self-renewal markers NANOG, OCT4 and SOX2 in tumour spheroids. IL-6/STAT3 triggered PIWI-interacting RNA 823 (piRNA 823) expression, which then promoted global DNA methylation via DNA methyltransferase 3β (DNMT3B) and increased the tumorigenic potential of MM cells. This phenotype was reversed by the addition of piRNA 823 antagomir or DNMT3B inhibitor, demonstrating that IL-6 can also enhance the stemness in cancer cells, hence treatment resistance of MM by inducing epigenetic changes (Ai et al., 2019).

Mutation of ten-eleven translocation 2 (TET2), an epigenetic regulator in haematopoietic stem and progenitor cells (HSPCs) is often observed in myeloid malignancies and results in increased self-renewal and myeloid proliferation in patients (Cimmino et al., 2017). Interestingly, the BM environment also plays a key role here. IL-6 induces hyperactivation of the Shp2–Stat3 signalling axis, resulting in increased expression of a long noncoding RNA, Morrbid, in TET2 mutant myeloid cells and HSPCs (Cai et al., 2018). Morrbid in turn represses transcription of the neighbouring pro-apoptotic gene, Bcl2l11 to promote survival of malignant cells in acute myeloid leukaemia (AML) and chronic myeloid leukaemia. These phenotypes are only observed in a subset of patients with TET2 mutations, suggesting that extrinsic factors IL-6 could possibly contribute to disease progression.

More importantly, since IL-6 transcription can be triggered by inflammatory cytokines including TGFβ, IL-1, and TNFα which are also activated mainly by NF-κB and STAT3, IL-6 within the BM microenvironment can create a feed-forward loop to propagate tumour progression. Also an osteoclast differentiation modulator which encourages osteoclastogenesis when bound to progenitors, dysregulation of IL-6 can result in excessive osteoclastic activity and osteolysis, a common hallmark observed in haematological malignancies (Steeve et al., 2004).

#### 1.3.2 TGFβ

Non-myelinating Schwann cells which encase peripheral nerves and lay in parallel with the BM vasculature (Yamazaki et al., 2011), as well as megakaryocytes, a cell type that physically associates with HSCs in the BM are one of the proposed sources of Transforming growth factor-β (TGFβ) (Bruns et al., 2014). TGFβ signals through a heteromeric receptor complex composed of two type-I and two type-II (TGFβRII) transmembrane serine-threonine kinase receptors, which then phosphorylate and activate TGFβRI. As a result, Smad2, and Smad3 are phosphorylated and form heterotrimers with Smad4. Smad2/Smad3/Smad4 complex translocates into the nucleus and interacts in a cell-specific manner with transcription factors to regulate the transcription of TGFβ target genes (Massagué et al., 2000; Figure 2).

TGFβ potentially regulates hematopoietic ageing and HSCs bias to myeloid lineage, with a decreased lymphoid potential in aged individuals, as shown by its role in expanding myeloid-biassed HSCs *in vitro* (Challen et al., 2010). Genetic deletion of Transcriptional Intermediary Factor 1 γ (TIF1γ), a novel regulator of TGFβ/SMAD signalling accelerated ageing increased expression of TGFβ receptors. This in turn leads to upregulated TGFβ/SMAD signalling and hence upregulation of the transcription factors such as RUNX1, which are known to regulate myeloid cell survival and differentiation of HSC (Himes et al., 2005; Quéré et al., 2014). Consistent with myeloid-bias in TGFβ role, loss of function of TGFβ signal is also reported in acute T cell lymphoblastic leukaemia (T-ALL), suggesting TGFβ-deficient haematopoietic cells shift to lymphoid lineage (Wolfraim et al., 2004). These findings suggest the role of aberrant TGFβ signalling in epigenetically modifying the BM microenvironment and thereby enhancing HSC ageing and myeloid-bias, promoting disease progression in myeloid malignancies.

#### 1.3.3 IL-17

IL-17, originally discovered in T cell hybridoma, is a proinflammatory cytokine. IL-17 is secreted by activated T helper 17 lymphocytes such as CD4 Th17 and CD8 Tc17 cells, while the IL-17 receptor complex is ubiquitously expressed (Rouvier et al., 1993). IL-17 receptor signalling recruits the adaptor molecule Act1, which in turn recruits tumour necrosis factor-R-associated factor 6 (TRAF6). Subsequently, TRAF6 recruits the kinase TAK1 and its binding partners TAB2 and TAB3 which activate the inhibitor of NF-κB kinase (IKK) complex, thereby inducing NF-κB translocation and activation or repression of target genes (Figure 2). It can also activate the JAK/STAT3 pathway in a similar manner (Gaffen, 2009).

Although the pro-inflammatory milieu sounds hostile to cancer cells, IL-17 orchestrates a cancer-supportive microenvironment, where the key player is neutrophils. γδT cells secrete IL-17, which recruits and expands neutrophils in the tumour, where the neutrophils suppress cytotoxic CD8^+^ T cells and promote metastasis of cancer. Blocking IL-17 was shown to reduce both neutrophils and cancer metastasis in mouse models (Coffelt et al., 2015). In T-ALL, an aggressive malignancy derived from early T cell progenitors (Raetz and Teachey, 2016), it has been observed that there is an upregulation of IL-17A in patients. Moreover, in IL17A^−/−^ mice models, IL17A activation upregulated Matrix metallopeptidase 9 (MMP9) expression via the NF-κB pathway, conferring the ability of degrading the extracellular matrix and promoted T-ALL cell metastasis to the liver (Tu et al., 2019). In addition, IL-17 in T-ALL also induces the production of different inflammatory cytokines such as IL-6, TNF-α and prostaglandins, which further favours the progression of the disease by endowing stemness and self-renewal in cancer cells, allowing them to evade immune response, and acquire chemoresistance (Ankathatti Munegowda et al., 2011). Altogether, IL-17 facilitates a cancer-supportive microenvironment through inflammatory programs.

#### 1.3.4 TNF-α

Tumour necrosis factor-α (TNFα) is an inflammatory cytokine mainly produced by activated macrophages, limited quantities by T lymphocytes and natural killer (NK) cells. It exists in two bioactive forms: the transmembrane (tmTNF-α) and secretory forms (sTNF-α), where sTNF-α is released from tmTNF-α via proteolytic cleavage by the metalloprotease TNF-α-converting enzyme (Black et al., 1997). The sTNF-α binds to either TNFR1 expressed on most mammalian cells, or TNFR2 primarily expressed on HSCs (Aggarwal, 2003). TNFα regulates pro-inflammatory responses and homeostatic processes such as cell communication, differentiation and apoptosis through caspase-mediated apoptosis pathways, MAPK signalling (ERK, JNK, p38α), or NF-κB signalling pathways (Enesa et al., 2008; Figure 2).

In myeloid leukaemia and MDS, sTNF-α secreted by the CSCs (autocrine) or by macrophages (paracrine) during chronic inflammation could promote NF-κB pathway/p65 signalling pathway, leading to rapid downregulation of pro-apoptotic genes CASP-8 and upregulation of pro-survival gene Birc3. As a result, this supports HSCs survival by inhibiting cell-cycle activators, thus re-establishing quiescence (Bruns et al., 2014; Yamashita and Passegué, 2019) Persistent TNF-α-driven chronic inflammation contributes to malignant haematopoiesis and therefore treatment resistance.

## 2 Clinical implications

### 2.1 Epigenetic therapies-challenges of monotherapy

Contrary to genetic mutations, epigenetic alterations are highly dynamic and flexible which involves reversible enzymatic reactions, which make them subjectable to pharmacological interventions. Epigenetic drugs currently approved by the FDA are DNA methyltransferase (DNMT) inhibitors for the treatment of MDS and AML, in combination with the BCL-2 inhibitor venetoclax for AML, and histone deacetylase (HDAC) inhibitors for the treatment of relapsed MM and cutaneous/peripheral T cell lymphoma. The principle of epigenetic drugs is to reprogramme the epigenetics of cancer cells and revert the self-renewal, stem-like phenotype of cells, thereby inducing differentiation towards a non-malignant phenotype. Theoretically, they could block invasion or metastasis of malignant cells from the BM to other tissues. However, initial studies on the general use of DNMT inhibitors and HDAC inhibitors were unfavourable (West and Johnstone, 2014). Azacitidine, a DNMT inhibitor failed to fully eradicate the leukaemic progenitor cell populations in patients with AML and MDS (Craddock et al., 2013), and decitabine, also a DNMT inhibitor only achieved in complete remission in 14% of MDS, 7% of AML and 10% of CMML patients (Helbig et al., 2019). Final results of a pivotal study on romidepsin, an HDAC inhibitor in relapsed/refractory CTCL also only showed an overall response rate of 34% in patients with prior chemotherapy romidepsin (Whittaker et al., 2010). Hence, epigenetic drugs, though in theory could revert the malignant and stem-like phenotype in cells of haematological malignancies, does not show promising translational efficacy as a monotherapy. It currently only remains as a palliative treatment instead of a cure.

### 2.2 Combination therapy

In recent years, immunotherapy, particularly immune checkpoint inhibitors (ICIs) are a hot spot in treating haematological malignancies which solves many issues seen in traditional cancer treatment. Although immunotherapy is promising, resistance acquisition and tumour escape via mechanisms such as loss or downregulation of antigen presentation (MHC), co-stimulation (CD80, CD86), and/or adhesion molecules, are still common issues among patients (Dustin, 2014; Roemer et al., 2016; Christopher et al., 2018).

Current excitement targeting cytokine production in the BM microenvironment to curb haematological malignancies has resulted in a number of ongoing clinical trials. Targeting production of the aforementioned cytokines theoretically should curtail and even reverse stemness of cancer cells. However, little success has occurred clinically. For example, siltuximab, an anti-IL-6 monoclonal antibody alone did not improve progression free survival (PFS) or overall survival (OS) in two separate Phase 2 clinical trials for patients with relapsed or refractory MM (Voorhees et al., 2013; NCT00401843). TNF-α monoclonal antibodies such as Etanercept (Enbrel), Infliximab (Remicade) and adalimumab (Humira) which are approved by the FDA for treatment of autoimmune diseases (Olsen and Stein, 2004) also had limited efficacy in treating hematologic malignancies in early clinical trials (Tsimberidou and Giles, 2002). This is because the currently FDA-approved TNF-α antibodies bind to both sTNF-α and tmTNF-α (Scallon et al., 2002). Despite improving haematopoiesis in MDS patients by increasing platelet and neutrophil counts (Stasi and Amadori, 2002; Raza et al., 2004), the targeted binding of cancer cells are mostly neutralised by sTNF-α, thereby lowering treatment efficacy.

Theoretically, antagonists of such cytokines can improve antigen priming and reinvigorate effector lymphocytes in haematological malignancies (Chen and Mellman, 2017). However, primary and acquired resistance mechanisms, as well as lack of fine-tuned antagonists significantly limit the patient subsets who can respond to these antagonists as monotherapies.

Successful immunotherapy responses in cancer patients often relies on a high accumulation of tumour-infiltrating lymphocytes (TILs) which makes cancer cells more susceptible to type 1 T helper cell IFN-γ-mediated anti-tumour immune responses (Zipeto et al., 2016). Type I IFN can activate several immune cell types such as dendritic cells, NK cells, and CD8^+^ T cells, whilst dampening the immunosuppressive actions of regulatory T cells and MDSCs (Dustin, 2014). Interestingly, evidence shows epigenetic drugs not only extensively modifies the tumour epigenome in haematological malignancies, it also rewires the chromatin landscape of immune cells in the BM microenvironment, in turn modulating the extent and quality of anti-tumour immune responses (Peng et al., 2015). Since epigenetic modification is a common hallmark seen in both immune cells and tumour cells within the BM microenvironment to confer drug resistance, epigenetic drugs can modulate such key regulatory features in both sectors and overcome some current limitations of immunotherapy.

For instance, entinostat, an HDACi increased the level of MHC class II-mediated antigen presentation by transcriptional activation of Class II Major Histocompatibility Complex Transactivator (CIITA) in diffuse large B cell lymphoma (DLBCL) (Cycon et al., 2013). Moreover, Tiper and Webb also demonstrated that Trichostatin-A, a pan-HDACi enhanced both CD1d- and MHC class II-mediated antigen presentation in mantle cell lymphoma (MCL), as well as inhibiting STAT3-regulated inflammatory cytokine secretion by MCL cells (Tiper and Webb, 2016). These preclinical data show the potential role of epigenetic drugs in alleviating tumour escape and increasing therapeutic success.

In light of this, various combination therapies of immune checkpoint inhibition and epigenetic therapy have emerged lately, aiming to achieve a higher therapeutic efficacy. An example of the combination of epigenetic drugs and ICIs in haematological malignancies is the use of azacytidine and magrolimab in AML. Prophagocytic signals such as CD47 on AML cells can be induced by cell damage after treatment with chemotherapy or epigenetic drugs avoid phagocytosis, and magrolimab, an anti-CD47 antibody has been under investigation in the treatment of myeloid malignancies after chemotherapy (Jaiswal et al., 2009). CD47 is upregulated on circulating hematopoietic stem cells and leukaemia cells to avoid phagocytosis. However, magrolimab alone is not effective enough to eradicate AML cells due to antigen escape (Majeti et al., 2009). The same group later investigated the combination approach with azacytidine, and preclinical data showed that azacitidine induced a 4 to 6-fold increase in CD47 expression in AML cells *in vitro*. Combination of azacitidine and magrolimab led to significantly higher macrophage-mediated phagocytosis of AML cells *in vitro* compared to single agents alone (Chao et al., 2020), and this therapy is currently undergoing its phase 1b clinical trial (NCT03248479), with the most recent data updated in December 2019. 73% of AML patients and 44% of MDS patients who were transfusion-dependent became red blood cell transfusion independent after treatment., with a safety profile similar to that observed in azacitidine monotherapy (Sallman et al., 2019).

Pembrolizumab, an anti-PD1 antibody and romidepsin, an HDAC inhibitor for T cell lymphoma is also undergoing its phase I/II clinical trial. Though no results related to T cell lymphoma patients have been posted as of now, preclinical data showed that HDAC inhibition increased levels of T cell chemoattractants and MDSC infiltration in multiple lung adenocarcinoma models. This is correlated to the sensitisation through anti-PD-1, showing promising potential of this combination therapy (Zheng et al., 2016).

Multiple ongoing clinical trials are using this combination strategy of epigenetic drugs and immune checkpoint inhibitors in haematological malignancies such as follicular lymphoma (NCT03150329), Hodgkin lymphoma (NCT03250962), MDS or AML (NCT02890329). An overview of other combinations in haematological malignancies is shown in Table 2.

**TABLE 2:** Overview of combination therapies of haematological malignancies using epigenetic drugs and immune checkpoint inhibitors.

ClinicalTrials.gov identifier	Investigating party	Phase	Status	Cancer type	Immune checkpoint inhibitor(s)	Epigenetic drug(s)	Efficacy
NCT01238692	Jewish General Hospital	2	Completed	DLBCL	Rituximab	Oral Panobinostat (HDACi)	No improvement of outcome when combined with rituximab
NCT01282476	Massachusetts General Hospital	2	Terminated	Relapsed/Refractory DLBCL	Rituximab (anti-CD20 antibody)	Panobinostat	
NCT01686165	University of Arizona Cancer Centre	2	Completed	Relapsed aggressive (high-risk) non-Hodgkin lymphoma	Rituximab	Belinostat (HDACi) plus Yttrium-90 (radioactive isotope)	All patients progressed after receiving therapy
NCT03150329	City of Hope Medical Center	1	Active, not recruiting	Relapsed/Refractory DLBCL, Follicular Lymphoma/Hodgkin Lymphoma	Pembrolizumab (anti-PD1 antibody)	Vorinostat (HDACi)	No results posted
NCT03250962	Chinese PLA General Hospital	2	Recruiting	Relapsed or Refractory Hodgkin Lymphoma after failed anti-PD1 antibody monotherapy	Camrelizumab (anti-PD1 antibody)	Decitabine (DNMTi)	CR: 79%
							Median PFS: 35.0 months
NCT02936752	National Cancer Institute	1b	Active, not recruiting	MDS following DNMTi therapy failure	Pembrolizumab (anti-PD1 antibody)	Entionstat (HDACi)	No results posted
NCT02845297	Sidney Kimmel Comprehensive Cancer Center at Johns Hopkins	2	Completed	Relapsed/refractory AML	Pembrolizumab	Azacytidine (DNMTi)	14% achieved CR, 10.8 months median OS
NCT02530463	M.D. Anderson Cancer Center	2	Active, not recruiting	MDS	Nivolumab (anti-PD1 antibody)	Azacytidine	No results posted
NCT02890329	National Cancer Institute	1	Active, not recruiting	Relapsed/refractory MDS/AML	Ipilimumab (anti-CTLA-4 antibody)	Decitabine	No results posted
NCT02508870	Hoffmann-La Roche	1	Completed	Relapsed/refractory MDS	Atezolizumab (anti-PD-L1)	Azacitidine	No improvement of outcome when combined with atezolizumab
NCT02281084	Celgene	2	Active, not recruiting	MDS following iHMA treatment failure	Durvalumab (anti-PD-L1 antibody)	Oral azacitidine	Progressive disease cohort median follow-up OS: 8.35 months in azacitidine alone group; 13.33 months in combination group
NCT02117219	MedImmune LLC	1	Completed	MDS	MEDI4736 (anti-PD-L1 antibody) + Tremelimumab (anti-CTLA-4 antibody)	Azacitidine	No results posted
NCT02775903	Celgene	2	Completed	MDS, AML	Durvalumab	Azacitidine	No improvement of outcome when combined with durvalumab
NCT03248479	Gilead Sciences	1b	Active, not recruiting	relapsed/refractory AML and MDS	Magrolimab (anti-CD40 antibody)	Azacitidine	No results posted

Note: All clinical trial information was obtained from ClinicalTrials.gov as of June 2023. Abbreviations: CR, complete remission; DLBCL, Diffuse Large B cell Lymphoma; DNMTi, DNA, methyltransferase inhibitor; HDACi, Histone deacetylase inhibitor; iHMA, injectable hypomethylating agent; MDS, myelodysplastic syndrome; OS, overall survival; PD1, programmed death-protein 1; PD-L1, programmed death-ligand 1; PFS, progression-free survival.

However, it is of note that two DLBCL clinical trials (NCT01238692, NCT01282476) reported no improvement in clinical outcome when panobinostat, an HDACi is combined with rituximab, an anti-CD20 antibody. This highlights the importance of optimising response-predicting biomarkers for combination therapies in order to screen for patient subsets likely to respond and make rational-based treatment choices. With most clinical trials currently being in their early phases, it is too early to know whether clinically significant efficacy will emerge from these ongoing trials to warrant movement of these combination therapies towards clinical use. Nevertheless, preclinical data highlights the importance of the immune-epigenetic crosstalk in haematological malignancies. Screening for epigenetic signatures using global strategies such as exome sequencing for unbiased mutation discovery in plasma could potentially serve as ideal biomarkers.

## 3 Conclusion

Today, drug resistance remains a crucial obstacle to the treatment of most haematological malignancies. It is a multifaceted process due to intracellular mechanisms such as intrinsic signalling pathways within the cancer cells due to mutations, extrinsic factors such as soluble factors and cell-cell interactions in the BM microenvironment which serves as a ‘tumour haven’. Activation of their respective signalling pathways within cancer cells induce epigenetic changes or secondary mutations in cancer cells and in turn promote tumour cell survival, self-renewal and drug resistance (Gajewski et al., 2006; Tredan et al., 2007; Meads et al., 2008). The BM microenvironment in haematological malignancies is highly dynamic and heterogeneous, comprising cancer cell subsets each with different epigenetic modifications involved, depending on the inflammatory state. Therefore, epigenetic monotherapy as a one-fits-for-all drug is not effective to fully eradicate malignant cells or revert their malignant phenotypes. As focused on this review, combining epigenetic therapy with ICI is, therefore, a possible combinatorial approach to enhance treatment efficacy in relapsed patients.

Understanding the intricate differences between normal and malignant niches, as well as the immune-epigenetic crosstalk within the BM microenvironment and their involvement in the phenotypic switch of cancer cells, is therefore needed to provide insights into developing novel therapies. As the results of the aforementioned clinical trials are being reported in the coming years, a focus on optimising biomarkers for these combination therapies will be quintessential to allocating each one of them to the correct patient subtypes.
